# Real-Time Monitoring and Predictive Analytics for Preventing and Reducing Restrictive Practices in Health and Social Care: Protocol for a Scoping Review

**DOI:** 10.2196/98754

**Published:** 2026-11-05

**Authors:** Angela Rong Yang Zhang, June Alexander, Lijun Zhao, Jennifer Tieman, Priyanka Vandersman, Donald Dong Zhang, Russell S A Brinkworth, Niranjan Bidargaddi, Shengjian Jammy Chen

**Affiliations:** 1 Research Centre for Palliative Care, Death and Dying, College of Health and Enablement, Flinders University, Bedford Park, South Australia, Australia; 2 Disability and Community Inclusion Unit, College of Health and Enablement, Flinders University, Bedford Park, SA, Australia; 3 Digital Health, College of Medicine and Public Health, Flinders University, Bedford Park, SA, Australia; 4 Research Centre for Palliative Care, Death and Dying, College of Health and Enablement, Flinders University, GPO Box 2100, Adelaide, South Australia, SA 5001, Australia, 61 8 7221 8237; 5 Wireless Systems and Security, College of Science and Engineering, Flinders University, Tonsley, South Australia, Australia; 6 Autonomous Systems, College of Science and Engineering, Flinders University, Tonsley, SA, Australia

**Keywords:** physical restraint, coercion, patient monitoring, digital health, AI, machine learning, clinical decision support systems, long-term care, mental health services, persons with disability

## Abstract

**Background:**

Restrictive practices, including restraint and seclusion, are used across aged care, mental health, and disability support settings to manage behaviors of concern. These practices are associated with substantial ethical, psychological, and physical harms. Digital health technologies, including real-time monitoring systems and AI-enabled predictive analytics, are increasingly used to detect early behavioral escalation. However, detection or prediction alone does not reduce restrictive practices. It remains unclear how these technologies are translated into care actions, decision-making, and measurable outcomes in real-world settings.

**Objective:**

This scoping review aims to identify and map evidence on real-time monitoring, predictive analytics, and related digital technologies used to prevent or reduce restrictive practices in health and social care. The review will examine the technologies and contexts studied; how technology outputs are translated into care responses and workflows; and the implementation, outcomes, governance, and ethical considerations reported.

**Methods:**

The review will be conducted in accordance with Joanna Briggs Institute methodology and will be reported using the PRISMA-ScR (Preferred Reporting Items for Systematic Reviews and Meta-Analyses Extension for Scoping Reviews). Librarian-assisted searches were conducted in MEDLINE, CINAHL Ultimate, PsycINFO, Scopus, and IEEE Xplore on March 11, 2026. The executed database search strategies were not modified after Open Science Framework registration. Reference lists of included studies and relevant reviews will be screened using citation chasing. Eligible English-language sources will be those that examine digital technologies linked to care responses intended to reduce restrictive practices or to reported restrictive-practice outcomes. Two reviewers will independently screen records and chart all included sources. Standard scoping-review mapping categories will be used to describe the evidence landscape. The Implementation Research Logic Model will inform analysis of explicitly reported implementation determinants, strategies, mechanisms, and outcomes.

**Results:**

Electronic database searches were completed on March 11, 2026. Protocol version 5.0 was registered with the Open Science Framework on March 22, 2026. Title and abstract screening had not commenced at the time of registration. The review is expected to be completed by July 2027.

**Conclusions:**

By clarifying how digital detection and prediction technologies are translated into care actions, and the conditions under which restrictive practice–related outcomes are reported, this review will support safer, more ethical, and more implementable digital health approaches.

## Introduction

### Background

Reducing restrictive practices affecting people who display behaviors of concern in health and social care is an international policy, human rights, and quality-of-care priority [1,2]. The following scenario illustrates a common pattern in health and social care settings. In a residential aged care facility, a person living with advanced dementia becomes increasingly distressed, paces repeatedly, raises their voice, and resists routine care. Early signs are noticed informally but are not systematically recognized or escalated. As the situation intensifies, staff respond reactively, and physical restraint is used to manage perceived risk. A later review suggests that earlier recognition and less intrusive responses may have prevented the restraint. This scenario illustrates how delayed recognition and response may contribute to escalation and the subsequent use of restrictive intervention.

In this review, “behaviors of concern” refers to behaviors such as agitation, aggression, resistance to care, wandering, disruptive vocalizations, or self-injury that may place the person or others at risk, disrupt care routines, or compromise well-being [1]. This term is increasingly preferred over “challenging behaviors” in contemporary health and social care policy and practice.

Behaviors of concern occur across aged care, disability, and mental health settings. In aged care, they may be associated with dementia, cognitive decline, pain, distress, or unmet needs [2]. In disability services, they may reflect communication difficulties, sensory sensitivities, or unmet support needs. In mental health settings, they may express psychological distress or responses to environmental triggers [3]. Restrictive practices, such as physical restraint, seclusion, and chemical sedation, are frequently used to manage these behaviors in aged care, mental health [4,5], and disability settings [1,2,4].

Restrictive practices are interventions that restrict a person’s movement, liberty, access, or decision-making [6]. In aged care and related policy contexts, these practices commonly include chemical restraint, environmental restraint, mechanical restraint, physical restraint, and seclusion [7]. Examples include using medication primarily to control behavior, blocking access to a space, applying straps or devices, physically directing a person against their wishes, or confining a person in a room or area. These practices can cause injury, trauma, and death [8], underscoring the need for prevention-focused approaches.

International and national policies increasingly emphasize reducing or eliminating restrictive practices through person-centered, rights-based, and least-restrictive approaches [2,6,7]. Established nondigital frameworks such as Safewards [9] and positive behavior support [10] have been developed across mental health, disability, and aged care settings. These approaches typically emphasize early recognition, relational care, de-escalation, staff capability, and supportive organizational cultures [9,10]. Large evaluations and systematic reviews suggest that such programs can reduce restraint or seclusion when they are implemented with sufficient fidelity and sustained culture change [9,11-13].

Restrictive practices occur across multiple sectors. Comparable debates about the governance, proportionality, and oversight of restrictive practices arise in correctional settings; however, this review focuses specifically on health and social care contexts, where regulatory frameworks, ethical mandates, and care objectives differ substantially.

Digital health tools may complement nondigital care strategies by supporting continuous monitoring of selected behavioral, physiological, or care-related indicators [14-22]. Examples include bed-chair sensors, wearable devices, real-time positioning systems, motion-monitoring technologies, ambient sensors, and systems using observational or biometric data [15-22]. Across heterogeneous applications, these technologies have been investigated for ambient monitoring, assessment of agitation and other behaviors of concern, prediction of self-harm, identification of delirium, and analysis of routinely collected health data [23-31]. Their potential value lies not simply in detection but in whether they trigger timely, proportionate, and least-restrictive care responses.

Recent advances in AI have expanded monitoring and prediction beyond sensor-based systems to include multimodal data, speech, text, and other digital traces [32]. Speech-emotion recognition research has investigated whether deep-learning models can identify emotion-related vocal features in real time [33]. However, the relevance and safe applications of these approaches in health and social care remain to be established. AI-enabled emotion recognition and prediction raise substantial ethical concerns, including bias, misclassification, privacy, consent, and accountability [34]. These concerns are especially important when outputs may influence restrictive or coercive responses. For this reason, the review will examine not only technological functions but also how outputs are interpreted, governed, and integrated into care workflows.

However, the ability to detect or predict behavioral escalation does not, by itself, reduce restrictive practices. It remains unclear what care actions follow detection, how these actions are operationalized in real-world settings, and whether they reduce restraint, seclusion, or other restrictive-practice outcomes. This review therefore focuses on technologies explicitly connected to restrictive-practice prevention, reduction, management, or outcomes, rather than on general monitoring or risk-prediction systems alone.

Health and social care end users, policymakers, and funders increasingly expect digital health studies to articulate a clear, testable causal pathway from context to outcomes, consistent with contemporary guidance on the development and evaluation of complex interventions [35]. However, preliminary searches of relevant evidence-synthesis databases conducted during protocol development did not identify an existing or ongoing scoping review specifically examining how real-time monitoring and predictive analytics are translated into care responses and restrictive practice–related outcomes across health and social care settings. Existing studies often provide insufficient reporting of the links among contextual determinants, implementation strategies, mechanisms, and outcomes. To date, the evidence has not been systematically brought together to examine not only detection but also the subsequent actions taken and the restrictive practice–related outcomes reported.

This scoping review aims to examine these pathways to capture who a digital health innovation reaches, how it works in context, and why it delivers (or fails to deliver) the expected outcomes. To do this, the review will use the standard scoping-review categories to map source characteristics, populations, settings, restrictive-practice types, technologies, data sources, analytic approaches, technology outputs, care responses, and outcomes. The Implementation Research Logic Model (IRLM) [36] will be used selectively to organize explicitly reported relationships among implementation determinants, strategies, mechanisms, and outcomes. The review will not infer implementation processes or causal pathways that are not reported in the source literature.

This review is needed because, as digital monitoring technologies are increasingly considered for care environments, consolidated guidance is required on their relationship to care responses, restrictive-practice outcomes, and ethical safeguards. Without clear evidence, services may miss opportunities to use digital tools to prevent harm. They may also adopt technologies that increase surveillance, coercion, or inequity. A consolidated review can clarify which technologies are linked to least-restrictive care responses, what outcomes have been reported, and what safeguards are needed.

Given the anticipated heterogeneity of technologies, settings, and outcomes, a scoping review is appropriate for examining how detection or prediction is linked to reported care responses and outcomes. Preliminary searches of evidence synthesis databases did not identify an existing or ongoing scoping review focused on the intersection of digital monitoring technologies and restrictive-practice reduction. This indicates a need for systematic evidence mapping.

### Objectives

The objective is to identify and map evidence on real-time monitoring, predictive analytics, and related digital technologies in health and social care where these technologies are linked to care responses intended to prevent, reduce, manage, or avoid restrictive practices or to reported restrictive practice–related outcomes. The review will focus on how detection or prediction is embedded within everyday care responses, decision-making processes, and workflows that lead, or fail to lead, to reductions in restraint or seclusion.

### Review Questions

What evidence exists on the use of real-time monitoring, predictive analytics, and related digital technologies to reduce or prevent restrictive practices in health and social care settings?

- Subquestion 1 (Q1): What types of digital technologies, data sources, analytic approaches, care settings, populations, and restrictive-practice types have been studied in relation to the prevention, reduction, management, or outcomes of restrictive practices?
- Subquestion 2 (Q2): How are technology outputs, such as alerts, risk scores, classifications, predictions, dashboards, or recommendations, translated into care responses, decision-making processes, workflows, nonrestrictive alternatives, and reported implementation, service, clinical, or restrictive practice–related outcomes?
- Subquestion 3 (Q3): What implementation determinants, barriers, enablers, and ethical or human rights considerations are reported in relation to the development, implementation, governance, and use of these technologies?

## Methods

### Study Design

This scoping review will be conducted in accordance with Joanna Briggs Institute (JBI) methodology for scoping reviews. The protocol has been developed with reference to relevant items of the PRISMA-ScR (Preferred Reporting Items for Systematic Reviews and Meta-Analyses Extension for Scoping Reviews) [37], where applicable to the protocol stage. The completed review will be reported in accordance with PRISMA-ScR. Standard scoping review mapping categories will be supplemented by IRLM-informed implementation analysis, where relevant information is explicitly reported. In line with the review objective, the methods are designed to examine not only whether digital monitoring and predictive technologies are used but also how detection or prediction is translated into care actions, decision-making, and operational workflows.

### Protocol Registration and Search Chronology

The librarian-assisted database search strategies were finalized before being executed across MEDLINE, CINAHL Ultimate, PsycINFO, Scopus, and IEEE Xplore on March 11, 2026. Protocol version 5.0 was subsequently registered with the Open Science Framework (OSF) on March 22, 2026 [38]. This interval arose because the administrative completion of the OSF record occurred after the librarian-assisted strategies had been finalized and executed. Registration was therefore not prospective with respect to search strategy development or database search execution.

The database search syntaxes executed on March 11, 2026, were not modified after OSF registration. Original database search histories and outputs have been retained, and the complete executed search strategies are provided in Multimedia Appendix 1. Subsequent protocol changes arising from editorial and peer review are documented as amendments and are not represented as having been prospectively registered.

### Eligibility Criteria

#### Participants/Population

This review will include people in health and social care settings who may experience behaviors of concern that can lead to restrictive practices. This includes people living with dementia, people with intellectual disability, mental health service users, hospital patients, and aged care residents. Care staff, carers, practitioners, and service leaders will also be within scope when the intervention targets their decisions or responses to behaviors of concern.

#### Concept

The concept includes 3 linked elements: restrictive practices, digital technologies, and care responses or outcomes that connect them.

Restrictive practices in scope include physical restraint, mechanical restraint, chemical restraint, environmental restraint, and seclusion. Studies that use different terminology will be eligible if the described practice substantively restricts movement, choice, autonomy, access, or liberty in a health or social care context.

Eligible technologies include real-time monitoring systems, sensors, wearables, environmental monitoring, cameras, microphones, physiological trackers, dashboards, alert systems, decision-support tools, machine learning (ML) models, large language models (LLMs), early warning systems, and behavior-forecasting algorithms. LLMs and natural language processing (NLP) applications will be included only when they are explicitly linked to restrictive-practice prevention, reduction, management, care-response pathways, or restrictive practice–related outcomes.

To be eligible, a study must explicitly link the technology to restrictive-practice prevention, reduction, management, avoidance, a care-response pathway, or a restrictive practice–related outcome. Studies in which technology outputs stop at detection or prediction alone will not be eligible unless the study explains how those outputs inform care responses or outcomes.

The review will also examine organizational, technological, ethical, workforce, and policy factors that affect implementation, uptake, and sustainability.

#### Context

The review will consider institutional, community, and home-based care settings in any country, including hospitals (mental health, emergency, and general), residential aged care, disability and group homes, and community and supported living services.

#### Types of Sources

This scoping review will consider peer-reviewed quantitative studies, qualitative studies, mixed methods studies, implementation evaluations, and pilot or feasibility studies that meet the inclusion criteria. Systematic reviews that meet the eligibility criteria will be included as evidence sources when they provide relevant information about technologies, care-response pathways, implementation processes, or outcomes. Primary studies already represented in included systematic reviews will be charted separately only where they independently meet the eligibility criteria and provide additional relevant data. When multiple reports describe the same study, intervention, or implementation program, reports will be linked, and duplicate outcome information will not be double-counted. Opinion pieces and editorials will be excluded unless they provide sufficient methodological or conceptual detail to determine whether the technology meets the minimum inclusion threshold.

#### Exclusion Criteria

Correctional and juvenile justice facilities will be excluded unless the intervention is primarily delivered in a health or social care context. No geographic exclusions will be applied. Searches will be limited to English-language sources published from 2000 onwards to capture developments in digital technologies. Sources not available in English will be excluded. Technology studies will be excluded where outputs stop at detection or prediction and do not explain how those outputs inform restrictive-practice prevention, reduction, management, avoidance, care responses, or related outcomes. The full inclusion and exclusion criteria, including the minimum inclusion threshold and restrictive-practice scope, are provided in Multimedia Appendix 2.

### Information Sources

Electronic database searches will include MEDLINE, CINAHL Ultimate, PsycINFO, Scopus, and IEEE Xplore. Reference lists of included studies and relevant reviews will be checked through backward citation searching, hereafter referred to as citation chasing. Any additional potentially eligible records identified through citation chasing will undergo the same screening and eligibility process.

### Search Strategy

A 3-step, JBI-aligned search strategy was used. First, an initial limited search of MEDLINE was conducted to identify relevant keywords and index terms. Second, librarian-assisted search strategies were developed and executed across MEDLINE, CINAHL Ultimate, PsycINFO, Scopus, and IEEE Xplore on March 11, 2026. Third, reference lists of included studies and relevant reviews were screened for citation chasing. The complete executed search histories document the databases, platforms, search dates, years covered, limits applied, search syntax, and numbers of records retrieved. These histories are provided in Multimedia Appendix 1.

### Source Selection

All identified citations will be exported to EndNote 20 (Clarivate) [39], and duplicates will be removed. Following a pilot test, 2 reviewers will independently screen titles and abstracts against the inclusion criteria. Potentially relevant sources will be retrieved in full and assessed in Covidence [40]. Reviewers will assess whether each source describes both a relevant technology and an explicit link to restrictive-practice prevention, reduction, management, avoidance, care responses, or outcomes. Disagreements will be resolved through discussion or, where required, adjudication by a third reviewer. Reasons for exclusion at full-text screening will be recorded. Final study selection will be reported using a PRISMA (Preferred Reporting Items for Systematic Reviews and Meta-Analyses) 2020 flow diagram adapted for the completed scoping review. Reference lists of included studies and relevant reviews will then be screened as part of citation chasing, and any additional potentially eligible records will undergo the same screening and eligibility process.

### Data Extraction and Charting

Data will be charted using a structured form in Multimedia Appendix 3. The form will be piloted with 5 studies and refined as necessary. The charting form is designed to accommodate heterogeneous reporting and will not require every field to be populated for every source. Missing information will be sought by contacting study authors when feasible.

Extracted data will include citation details, study characteristics, care setting, population, technology type, data source, target behaviors, restrictive-practice type, technology output, link to care response, implementation determinants and strategies, outcomes, and ethical considerations. Additional fields will capture whether and how technology outputs inform staff action, care planning, de-escalation, environmental adjustment, medication review, clinical escalation, or other responses intended to prevent, reduce, manage, or avoid restrictive practices.

Implementation strategies will be coded using the Expert Recommendations for Implementing Change taxonomy [41] where sufficient detail is available. Determinants will be mapped to IRLM categories, and ethical considerations will include privacy, consent, bias, surveillance harms, coercion, autonomy, equity, governance, and human rights framing.

Two reviewers will independently chart data, with disagreements resolved through discussion or adjudication by a third reviewer. Any substantive refinements to the charting form or codebook will be documented and applied consistently across all included sources.

### Critical Appraisal

The methodological quality of included empirical sources will be appraised using tools appropriate to the study design. The Mixed Methods Appraisal Tool (MMAT) [42] will be used for qualitative, quantitative, and mixed methods studies, where applicable. Relevant JBI critical appraisal tools [43] will be used for study types not adequately covered by the MMAT. Appraisal findings will support the interpretation of the evidence but will not be used to exclude sources.

### Synthesis of Results

Data will be collated, summarized, and reported in line with JBI guidance and PRISMA-ScR. The synthesis will be structured around the 3 review subquestions. Q1 will map the evidence landscape, including technologies, data sources, analytic approaches, settings, populations, and restrictive-practice types. Q2 will examine how technology outputs are translated into care responses, decision-making processes, workflows, nonrestrictive alternatives, and reported outcomes. Q3 will synthesize implementation determinants, barriers, enablers, and ethical or human rights considerations. Together, these strands will address the primary question of how real-time monitoring and predictive analytics are used to reduce or prevent restrictive practices in health and social care.

### Analytical Framework

The analysis will use the standard scoping-review categories and the IRLM lens. When multiple reports describe the same study, intervention, or implementation program, those reports will be linked by study identifiers, setting, technology, population, and implementation characteristics.

Extracted data will be categorized into 3 groups of variables aligned with the review subquestions. Q1 variables will map the evidence landscape, including study identifiers, population, country, setting, study design, restrictive-practice type, technology type, monitoring or predictive function, data source, technology output, and target behaviors. Q2 variables will trace what happens after detection or prediction, including care-response pathways, human decision points, decision rules, automation level, implementation strategies, mechanisms of action, and reported restrictive-practice, service, clinical, or implementation outcomes. Q3 variables will classify contextual determinants, barriers, enablers, and ethical or human rights considerations, including privacy, consent, surveillance harms, coercion, stigma, bias, autonomy, equity, governance, and the potential normalization of restrictive practices. Together, these variables will support descriptive statistics and logic-pathway summaries linking determinants, implementation strategies, mechanisms, care responses, and outcomes.

### Quantitative Summarization and Qualitative Synthesis

Quantitative data will be summarized descriptively using counts, proportions, medians, IQRs, and, where appropriate, cross-tabulations. Descriptive summaries will be organized around Q1, Q2, and Q3. Q1 summaries will describe the distribution of evidence by technology type, data source, care setting, population, and restrictive-practice type. Q2 summaries will describe care-response pathways, workflow integration, implementation strategies, mechanisms, and reported implementation, service, clinical, or restrictive practice–related outcomes. Q3 summaries will describe reported determinants, barriers, enablers, and ethical or human rights considerations. Meta-analysis and inferential effect estimation are not planned.

Qualitative synthesis will use framework-based thematic synthesis. Coding will begin deductively using IRLM domains and the Q1/Q2/Q3 structure, followed by inductive subthemes where needed. Where useful, findings will be integrated through logic-pathway summaries and narratives or visuals.

### Data Presentation

Extracted data will be presented in tabular and diagrammatic formats aligned with the review objectives. A descriptive numerical summary will outline the distribution of evidence by year, country, setting, and technology type. A narrative synthesis will summarize key themes regarding intervention characteristics, outcomes, and implementation enablers and barriers. Evidence gaps will be highlighted to inform future research directions.

### Stakeholder Consultation

Consistent with JBI guidance, a targeted stakeholder consultation approach will be used to enhance the relevance, interpretability, and ethical sensitivity of the review. Members of the Flinders University Inclusive Innovation Action Group, including lived-experience experts and service provider representatives, will be invited to provide advisory input at 2 stages.

First, stakeholders will provide feedback on the review questions, definitions of restrictive practices, and framing of postdetection care actions through a structured asynchronous format, such as written feedback via email or an online survey. Second, stakeholders will be invited to comment on a summary of preliminary findings to support the interpretation of implementation pathways, care workflows, and ethical considerations. Stakeholder input will contextualize findings and identify potential gaps or misinterpretations, but will not determine study inclusion, data extraction, or synthesis decisions. All stakeholder contributions will be acknowledged and reported transparently in the final publication.

### Protocol Amendments

Amendments arising from editorial and peer review included restriction to English-language sources, removal of gray-literature searching, clarification of the minimum inclusion threshold and LLM/NLP eligibility, revision of the independent data-charting procedure, clarification of systematic-review and overlapping-report handling, and removal of Center for eHealth Research and Disease Management (CeHRes) as a formal analytical framework. These amendments did not modify the database search syntaxes executed on March 11, 2026.

### Ethical Considerations

Ethics approval is not required because this scoping review will use publicly available literature and will not involve human participants. However, the topic has substantial ethical and human rights implications. Real-time monitoring, predictive analytics, and AI-enabled decision-support systems may introduce or amplify risks related to surveillance, coercion, bias, misclassification, privacy, consent, autonomy, equity, and governance. These risks are particularly important in settings where people may be subject to restrictive practices or may have limited power to refuse monitoring or challenge data-informed decisions.

To support ethically responsible synthesis, the charting form will capture reported safeguards, accountability mechanisms, human oversight, least-restrictive principles, and rights-based care responses. Technologies will therefore be considered not only in terms of detection or prediction but also in relation to dignity, autonomy, prevention, and transparent governance.

## Results

### Current Status

At the time of protocol submission, electronic database searches have been completed, with the final search conducted on March 11, 2026. The searches retrieved 828 records from MEDLINE, 88 from CINAHL Ultimate, 136 from PsycINFO, 482 from Scopus, and 24 from IEEE Xplore. A protocol-stage PRISMA 2020 flow diagram adapted for this scoping review is provided in Figure 1. Title and abstract screening, citation chasing, full-text eligibility assessment, and final inclusion decisions had not commenced and will be reported in the completed scoping review.

**Figure 1.**
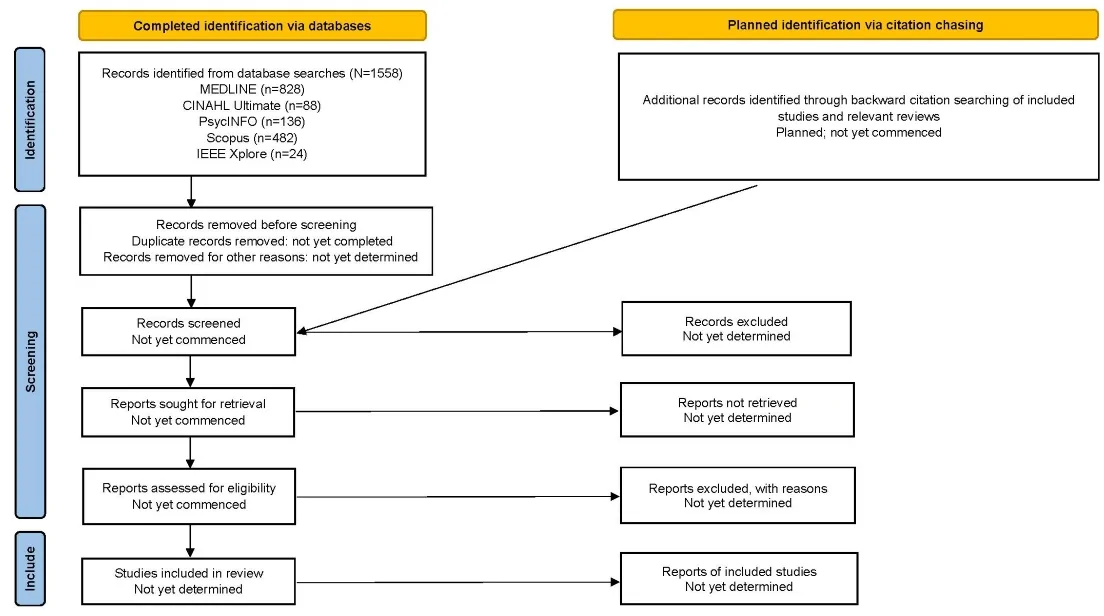
Protocol-stage PRISMA (Preferred Reporting Items for Systematic Reviews and Meta-Analyses) 2020 flow diagram adapted for this scoping review. Database searches were completed on March 11, 2026, and identified 1558 records across MEDLINE, CINAHL Ultimate, PsycINFO, Scopus, and IEEE Xplore. Duplicate removal, title and abstract screening, citation chasing, full-text eligibility assessment, and final inclusion decisions had not commenced when this protocol-stage figure was prepared and will be reported in the completed scoping review. Adapted from the PRISMA 2020 flow diagram for new systematic reviews—including searches of databases, registers, and other sources—by Page et al [44], under the Creative Commons Attribution 4.0 International License [45].

### Timeline and Expected Completion

Title and abstract screening is expected to commence following publication of this protocol, with full-text screening and data charting proceeding sequentially. The scoping review is expected to be completed in July 2027, with findings disseminated through peer-reviewed publications, conference presentations, and open-access outputs.

## Discussion

### Anticipated Findings

As a scoping review, this study will not produce pooled effect estimates or test hypotheses. Instead, it will provide a descriptive synthesis of evidence on real-time monitoring and predictive analytics used to reduce or prevent restrictive practices in health and social care. By requiring an explicit link between technology and restrictive practice–related care responses or outcomes, the review will distinguish technologies that merely detect or predict risk from those embedded within prevention-focused care processes.

The anticipated outputs include an evidence map of technologies and contexts, a synthesis of postdetection and postprediction processes, a summary of reported outcomes, an implementation-focused analysis of barriers and enablers, and an ethical synthesis addressing privacy, consent, surveillance, autonomy, and algorithmic bias.

Findings will be presented using tables, diagrams, and narrative synthesis, including cross-tabulations and logic-pathway summaries linking contextual determinants, implementation strategies, mechanisms of action, and outcomes. Evidence gaps and areas of uncertainty will be highlighted to inform future intervention development, evaluation, and policy decision-making.

### Comparison With Prior Work

Prior work on reducing restrictive practices has focused largely on nondigital, multicomponent approaches, especially positive behavior support in disability and aged care and Safewards in mental health services. These frameworks emphasize early recognition of escalation, relational care, de-escalation, structured staff responses, and supportive organizational cultures. Prevention therefore relies mainly on human observation, clinical judgment, and team-based interventions embedded in routine care.

Digital health research has increasingly examined technologies that monitor, detect, or predict behaviors of concern. These include sensor-based systems, real-time location services, and predictive analytics applied to clinical or behavioral data. Existing syntheses often focus on technical feasibility, accuracy, or acceptability; they less often examine how alerts or predictions are embedded in care workflows, how they interact with established frameworks such as positive behavior support or Safewards, or whether they are associated with measurable reductions in restrictive practices.

To date, no scoping review has been identified that systematically maps how real-time monitoring and predictive analytics are integrated into preventive care processes aimed at reducing restraint or seclusion across health and social care settings. Existing reviews have not examined how digital detection or prediction technologies complement, substitute for, or potentially disrupt established human-centered frameworks for restrictive practice reduction. This scoping review addresses that gap by synthesizing evidence across digital health and restrictive practice literatures and applying an implementation-focused analytical lens. By combining standard scoping-review mapping with IRLM-informed implementation analysis, the review extends prior work by examining not only what technologies are used but also how they are embedded within care processes, aligned with preventive frameworks, governed ethically, and under what contextual conditions they are associated with reduced reliance on restrictive practices.

### Strengths and Limitations

This scoping review protocol has several strengths. First, it is grounded in established scoping review methodology and will be conducted in accordance with JBI guidance and reported using the PRISMA-ScR framework, ensuring transparency, reproducibility, and methodological rigor. The review adopts a comprehensive and systematic approach to mapping evidence across multiple health and social care settings, populations, and digital technology modalities, allowing for a broad and inclusive examination of real-time monitoring and predictive analytics in relation to restrictive practice reduction.

Second, the review is strengthened by its explicit focus on postdetection and postprediction processes, moving beyond the question of whether technologies can identify risk to examine how alerts or predictions are operationalized into care actions, decision-making workflows, and outcomes. By using the IRLM, the review provides a structured, theory-informed analytical lens that enables systematic examination of contextual determinants, implementation strategies, mechanisms of action, and outcomes. This approach supports a nuanced understanding of why technologies may succeed or fail in reducing restrictive practices in real-world settings.

Third, the inclusion of a wide range of evidence sources, including quantitative, qualitative, and mixed methods studies, enhances the review’s ability to capture implementation, ethical, and governance considerations that are often underreported in controlled evaluations. The planned independent dual-reviewer screening and data-charting processes further strengthen methodological robustness and reduce the risk of selection and extraction bias.

Several limitations should also be acknowledged. As a scoping review, this study is not designed to assess intervention effectiveness or produce pooled effect estimates, and no meta-analysis is planned. Consequently, the review will describe the nature, distribution, and characteristics of the evidence rather than determine causal effects or comparative effectiveness of specific technologies. Although a methodological quality appraisal will be conducted to support interpretation, included sources will not be excluded on the basis of quality, which may result in the inclusion of heterogeneous or methodologically weak evidence. However, where low-quality or potentially flawed evidence is identified, it will be reported as such and given less weight in any conclusions.

The breadth of the review may also limit the depth with which individual technologies or settings can be examined, particularly given the diversity of care contexts, digital tools, and outcome measures. Variability in terminology, reporting standards, and outcome definitions across studies may further constrain comparability and synthesis. Finally, the review is limited to English-language sources identified through selected bibliographic databases and citation chasing. Relevant evidence outside these databases, in non-English sources, or in unpublished sources may therefore not be captured.

In addition, the database searches were executed before the administrative completion of OSF registration. Registration was therefore retrospective for search development and database identification, limiting prospective transparency and potentially introducing retrospective search bias. The review team has mitigated, but cannot eliminate, this limitation by reporting the chronology, retaining the original search histories and outputs, providing complete, executed search strategies, confirming that the syntax was not modified after registration, and documenting subsequent amendments.

Despite these limitations, the proposed scoping review is well suited to its primary aim of mapping existing evidence, identifying knowledge gaps, and clarifying how real-time monitoring and predictive analytics are translated into care actions and outcomes relevant to restrictive practice reduction.

### Dissemination Plans

Findings from the review will be published as open access in peer-reviewed journals and disseminated globally to targeted audiences across health and social care settings. Furthermore, plain-language summaries will be disseminated across conferences, social media, and digital platforms, reaching diverse audiences of policymakers, academic researchers, health and social care leaders, providers, and professionals.

### Conclusions

This scoping review will clarify how real-time monitoring, predictive analytics, and related digital technologies are translated into care actions intended to prevent or reduce restrictive practices. By mapping technologies, workflows, implementation determinants, outcomes, and ethical safeguards, the review will support more evidence-informed, rights-based, and implementable digital health approaches in health and social care.

## Supplementary material

10.2196/98754 Multimedia Appendix 1 Search strategies.

10.2196/98754 Multimedia Appendix 2 Eligibility criteria for digital technologies and restrictive practice–related care responses or outcomes.

10.2196/98754 Multimedia Appendix 3 Data extraction and charting form aligned with the 3 review subquestions.

10.2196/98754 Checklist 1 PRISMA-P checklist

## Abbreviations

CeHRes: Center for eHealth Research and Disease Management
IRLM: Implementation Research Logic Model
JBI: Joanna Briggs Institute
LLM: large language model
ML: machine learning
MMAT: Mixed Methods Appraisal Tool
NLP: natural language processing
OSF: Open Science Framework
PRISMA: Preferred Reporting Items for Systematic Reviews and Meta-Analyses
PRISMA-ScR: Preferred Reporting Items for Systematic Reviews and Meta-Analyses Extension for Scoping Reviews
Q1: subquestion 1
Q2: subquestion 2
Q3: subquestion 3
