# Supplementary material for: Real-Time Monitoring and Predictive Analytics for Preventing and Reducing Restrictive Practices in Health and Social Care: Protocol for a Scoping Review
Source: JMIR Res Protoc. 2026 Nov 5;15:e98754. doi: 10.2196/98754 (PMC13739262; doi:10.2196/98754)
Supplement: Supplementary file 1 [file resprot-v15-e98754-s001.docx]

Title

Real-time monitoring and predictive analytics to prevent and reduce restrictive practices in health and social care: a scoping review protocol

**Appendix II. Search Strategies**

**Search chronology and registration note:** The librarian-assisted strategies below were finalized and executed on March 11, 2026. Protocol version 5.0 was registered with the Open Science Framework on March 22, 2026. Administrative completion of the OSF record occurred after search execution. Comparison with the retained search histories confirmed that the executed database syntaxes were not modified after registration.

**MEDLINE**

**Information source: MEDLINE**

**Search platform:** Ovid

**Search date:** March 11, 2026

**Date range searched:** 2000 to current

**Limits and restrictions:** Animal-only records excluded; publication year limited to 2000 to current.

**Records retrieved:** 828 records

**Full search strategy**

1. Restraint, Physical/ or Coercion/ or Seclusion/ or Protective Devices/

2. ("restrictive practice*" or "restraint" or seclusion or isolation room* or coercion or "coercive practice*" or "forced treatment" or "restrictive intervention*" or "control intervention*" or "safety intervention*" or "use of force" or "chemical restraint*" or "mechanical restraint*" or "physical hold*" or "holding intervention*" or "liberty deprivation" or "freedom restriction" or involuntary treatment or "compulsory treatment" or "environmental restraint*" or "pharmacological restraint*" or "physical barrier restraint*").mp.

3. 1 or 2

4. Monitoring, Physiologic/ or Telemetry/ or Wearable Electronic Devices/ or Biosensing Techniques/ or Sensors/ or Remote Sensing Technology/ or Patient Monitoring/ or Biomedical Technology/ or Data Collection/ or Signal Processing, Computer-Assisted/ or Remote Consultation/

5. ("real-time monitoring" or "realtime monitoring" or "continuous monitoring" or "continuous observation" or "continuous assessment" or "remote monitoring" or telemonitoring or "digital monitoring" or wearable* or sensor* or "biometric monitoring" or "early warning system*" or "continuous surveillance" or "continuous tracking" or "patient monitoring" or "health monitoring system*" or "activity tracker*" or "tracking device*" or "computer assisted signal processing" or "remote consultation" or "Biomedical Technolog*" or "data collection").mp.

6. 4 or 5

7. Artificial Intelligence/ or Machine Learning/ or Deep Learning/ or Natural Language Processing/ or Neural Networks, Computer/ or Algorithms/ or Data Mining/ or Pattern Recognition, Automated/ or Decision Support Systems, Clinical/ or Predictive Value of Tests/ or Early Diagnosis/ or Risk Assessment/ or Models, Statistical/ or Models, Theoretical/ or Prognosis/ or Machine Learning Algorithms/ or Prediction Algorithms/

8. ("predictive analytic*" or "predictive model*" or "predictive algorithm*" or "predictive system*" or "risk prediction" or "risk forecasting" or "early warning system*" or "early detection algorithm*" or "forecasting model*" or AI or "artificial intelligence" or "machine learning" or ML or "deep learning" or DL or "neural network*" or "natural language processing" or NLP or "pattern recognition" or "data-driven model*" or "data mining" or algorithm* or "clinical decision support" or "decision support system*" or "statistical model*" or "prognostic model*" or "predictive test*").mp.

9. 7 or 8

10. Generative Artificial Intelligence/

11. ((Conversational or "embodied conversational" or artificial or "natural language") adj1 (agent or intelligence or processing)).mp.

12. (("Text?based" or virtual or relational or intelligent or synchronous or asynchronous or "user?computer" or "human?computer" or "computer assisted") adj2 (assistant or human or agent* or coach or chat* or person or people or interface or therap*)).mp.

13. ((anthropomorphic or automated or embodied or intelligent or virtual) adj3 (advisor or agent or assistant or consultant or expert or human or interface or machine or person or persona or people or representative or robot or specialist)).mp.

14. (chatgpt or chatbot or "large language model*" or "generative AI" or "Generative Artificial Intelligence").mp.

15. 10 or 11 or 12 or 13 or 14

16. 6 or 9 or 15

17. mental disorders/ or anxiety disorders/ or mood disorders/ or "schizophrenia spectrum and other psychotic disorders"/

18. Stress Disorders, Post-Traumatic/ or Mental Health/ or Bipolar Disorder/ or Depression/ or Mania/

19. ("psychiatric disorder*" or "mental disorder*" or "psychiatric condition*" or schizophrenia or "Post traumatic stress disorder" or "PTSD" or anxiety or "anxiety disorder*" or "psychotic disorder*" or "Mood disorder*" or "psychological trauma" or "Mental health" or "Mental illness" or "Mental disease" or "Psychiatric" or "Psychotic" or "Psychosis" or "Schizophre*" or "Bipolar disorder*" or "Depression" or "mania*" or "trauma inform*" or "trauma focus*" or "trauma based" or "trauma sensitive" or "trauma aware*").mp.

20. 17 or 18 or 19

21. exp animals/ not humans.sh.

22. exp Intellectual Disability/

23. ("Prader Willi syndrome" or "down syndrome" or "fetal alcohol syndrome" or "Fragile X" or "Williams Syndrome" or "Smith-Magenis Syndrome" or "Cri-du-chat Syndrome" or "Angelman Syndrome" or "Rett Syndrome" or "down syndrome").mp.

24. autism spectrum disorder/ or asperger syndrome/ or autistic disorder/ or communication disorders/ or learning disabilities/ or social communication disorder/ or speech sound disorder/ or developmental disabilities/

25. ("autism spectrum disorder" or "ASD" or "asperger syndrome" or autistic or "communication disorder*" or "learning disabilit*" or "social communication disorder" or "speech sound disorder" or "developmental disabilit*").mp.

26. brain injuries/ or brain injuries, traumatic/ or brain injury, chronic/ or Psychological Trauma/

27. ("Brain Injur*" or Psychological Trauma or PTSD or "post traumatic stress disorder").mp.

28. ((neurodevelopment or learning or communication or speech or motor or neuro) adj2 disorder*).mp.

29. Attention Deficit Disorder with Hyperactivity/

30. ("attention deficit disorder" or "ADHD").mp.

31. 22 or 23 or 24 or 25 or 26 or 27 or 28 or 29 or 30

32. dementia/ or alzheimer disease/ or lewy body disease/ or neurocognitive disorders/ or cognitive dysfunction/ or delirium/

33. (dementia or "Alzheimer disease" or "Lewy body disease" or "neurocognitive disorder*" or "cognitive dysfunction" or delirium).mp.

34. 32 or 33

35. 20 or 31 or 34

36. 3 and 16 and 35

37. 36 not 21

38. limit 37 to yr="2000 -Current"

Final set: 38 = 828 records.

**PsycINFO**

**Information source:** PsycINFO

**Search platform:** Ovid

**Search date:** March 11, 2026

**Date range searched:** 2000 to current

**Limits and restrictions:** Human limit applied; publication year limited to 2000 to current

**Records retrieved:** 136 records

**Full search strategy**

1. ("restrictive practice*" or restraint or seclusion or "isolation room*" or coercion or "coercive practice*" or "forced treatment" or "restrictive intervention*" or "safety intervention*" or "use of force" or "chemical restraint*" or "mechanical restraint*" or "physical restraint*" or "physical hold*" or "holding intervention*" or "liberty deprivation" or "freedom restriction" or involuntary treatment or compulsory treatment or "environmental restraint*" or "pharmacological restraint*" or "physical barrier restraint*").mp.

2. ("real-time monitoring" or "realtime monitoring" or "real time monitoring" or "continuous monitoring" or "continuous observation" or "continuous assessment" or "remote monitoring" or telemonitoring or "digital monitoring" or wearable* or sensor* or biosensor* or "biometric monitoring" or "patient monitoring" or "health monitoring system*" or "activity tracker*" or "tracking device*" or "continuous surveillance" or "continuous tracking").mp.

3. ("predictive analytic*" or "predictive model*" or "prediction algorithm*" or "predictive system*" or "risk prediction" or "risk forecasting" or "early detection algorithm*" or "forecasting model*" or "clinical decision support" or "decision support system*" or "prognostic model*" or "data mining" or "pattern recognition" or artificial intelligence* or "machine learning" or "deep learning" or "natural language processing" or "neural network*" or "large language model*" or "generative artificial intelligence" or chatbot* or "conversational agent*" or "embodied conversational agent*" or "virtual assistant*" or "AI chatbot" or "trained language model" or "relational agent*" or "virtual coach" or "digital assistant*" or "GPT" or "transformer model" or "intelligent assistant*").mp.

4. 2 or 3

5. ("psychiatric disorder*" or "mental disorder*" or "psychiatric condition*" or schizophrenia or "post traumatic stress disorder" or PTSD or anxiety or "anxiety disorder*" or "psychotic disorder*" or "mood disorder*" or "psychological trauma" or "mental health" or "mental illness" or psychiatric or psychotic or psychosis or schizoph* or "bipolar disorder*" or depression or mania* or "trauma informed" or "trauma inform*" or "trauma focused" or "trauma sensitive" or "trauma aware*").mp.

6. ("intellectual disabilit*" or "developmental disabilit*" or "Prader-Willi syndrome" or "down syndrome" or "fetal alcohol syndrome" or "Fragile X" or "Williams syndrome" or "Smith-Magenis syndrome" or "Cri-du-chat syndrome" or "Angelman syndrome" or "Rett syndrome" or "autism spectrum disorder" or ASD or "asperger syndrome" or autistic or "communication disorder*" or "learning disabilit*" or "social communication disorder" or "speech sound disorder" or "brain injur*" or "attention deficit disorder" or ADHD or "neurodevelopmental disorder*" or "neurodevelopment disorder*").mp.

7. (dementia or "Alzheimer disease" or "Lewy body disease" or "neurocognitive disorder*" or "cognitive dysfunction" or delirium).mp.

8. 5 or 6 or 7

9. 1 and 4 and 8

10. limit 9 to human

11. limit 10 to yr="2000 -Current"

Final set: 11 = 136 records

**CINAHL Ultimate**

**Information source:** CINAHL Ultimate

**Search platform:** EBSCOhost

**Search date:** March 11, 2026

**Date range searched:** 2000 to current

**Limits and restrictions:** Publication year limited to 2000 to current

**Records retrieved:** 88 records

**Full search strategy**

S1. TI ("restrictive practice*" OR restraint OR seclusion OR "isolation room*" OR coercion OR "coercive practice*" OR "forced treatment" OR "restrictive intervention*" OR "control intervention*" OR "safety intervention*" OR "use of force" OR "chemical restraint*" OR "mechanical restraint*" OR "physical restraint*" OR "physical hold*" OR "holding intervention*" OR "liberty deprivation" OR "freedom restriction" OR involuntary treatment OR "compulsory treatment" OR "environmental restraint*" OR "pharmacological restraint*" OR "physical barrier restraint*")

OR AB ("restrictive practice*" OR restraint OR seclusion OR "isolation room*" OR coercion OR "coercive practice*" OR "forced treatment" OR "restrictive intervention*" OR "control intervention*" OR "safety intervention*" OR "use of force" OR "chemical restraint*" OR "mechanical restraint*" OR "physical restraint*" OR "physical hold*" OR "holding intervention*" OR "liberty deprivation" OR "freedom restriction" OR involuntary treatment OR "compulsory treatment" OR "environmental restraint*" OR "pharmacological restraint*" OR "physical barrier restraint*")

S2. TI ("real-time monitoring" OR "realtime monitoring" OR "real time monitoring" OR "continuous monitoring" OR "continuous observation" OR "continuous assessment" OR "remote monitoring" OR telemonitoring OR "digital monitoring" OR wearable* OR sensor* OR biosensor* OR "biometric monitoring" OR "patient monitoring" OR "health monitoring system*" OR "activity tracker*" OR "tracking device*" OR "continuous surveillance" OR "continuous tracking")

OR AB ("real-time monitoring" OR "realtime monitoring" OR "real time monitoring" OR "continuous monitoring" OR "continuous observation" OR "continuous assessment" OR "remote monitoring" OR telemonitoring OR "digital monitoring" OR wearable* OR sensor* OR biosensor* OR "biometric monitoring" OR "patient monitoring" OR "health monitoring system*" OR "activity tracker*" OR "tracking device*" OR "continuous surveillance" OR "continuous tracking")

S3. TI ("predictive analytic*" OR "predictive model*" OR "prediction algorithm*" OR "predictive system*" OR "risk prediction" OR "risk forecasting" OR "early detection algorithm*" OR "forecasting model*" OR "clinical decision support" OR "decision support system*" OR "prognostic model*" OR "data mining" OR "pattern recognition" OR "artificial intelligence" OR "machine learning" OR "deep learning" OR "natural language processing" OR "neural network" OR "large language model*" OR "generative AI" OR "generative artificial intelligence" OR chatbot* OR "conversational agent*" OR "virtual assistant*" OR "digital assistant*" OR "relational agent*" OR "virtual coach" OR "intelligent assistant*")

OR AB ("predictive analytic*" OR "predictive model*" OR "prediction algorithm*" OR "predictive system*" OR "risk prediction" OR "risk forecasting" OR "early detection algorithm*" OR "forecasting model*" OR "clinical decision support" OR "decision support system*" OR "prognostic model*" OR "data mining" OR "pattern recognition" OR "artificial intelligence" OR "machine learning" OR "deep learning" OR "natural language processing" OR "neural network" OR "large language model*" OR "generative AI" OR "generative artificial intelligence" OR chatbot* OR "conversational agent*" OR "virtual assistant*" OR "digital assistant*" OR "relational agent*" OR "virtual coach" OR "intelligent assistant*")

S4. S2 OR S3

S5. TI ("psychiatric disorder*" OR "mental disorder*" OR "psychiatric condition*" OR schizophrenia OR "post traumatic stress disorder" OR PTSD OR anxiety OR "anxiety disorder*" OR psychotic disorder* OR "mood disorder*" OR "psychological trauma" OR "mental health" OR "mental illness" OR psychiatric OR psychotic OR psychosis OR schizoph* OR "bipolar disorder*" OR depression OR mania* OR "trauma informed" OR "trauma inform*" OR "trauma focused" OR "trauma sensitive" OR "trauma aware*")

OR AB ("psychiatric disorder*" OR "mental disorder*" OR "psychiatric condition*" OR schizophrenia OR "post traumatic stress disorder" OR PTSD OR anxiety OR "anxiety disorder*" OR psychotic disorder* OR "mood disorder*" OR "psychological trauma" OR "mental health" OR "mental illness" OR psychiatric OR psychotic OR psychosis OR schizoph* OR "bipolar disorder*" OR depression OR mania* OR "trauma informed" OR "trauma inform*" OR "trauma focused" OR "trauma sensitive" OR "trauma aware*")

S6. TI ("intellectual disabilit*" OR "developmental disabilit*" OR "Prader-Willi syndrome" OR "down syndrome" OR "fetal alcohol syndrome" OR "Fragile X" OR "Williams syndrome" OR "Smith-Magenis syndrome" OR "Cri-du-chat syndrome" OR "Angelman syndrome" OR "Rett syndrome" OR "autism spectrum disorder" OR ASD OR "asperger syndrome" OR autistic OR "communication disorder*" OR "learning disabilit*" OR "social communication disorder" OR "speech sound disorder" OR "brain injur*" OR "attention deficit disorder" OR ADHD OR "neurodevelopmental disorder*" OR "neurodevelopment disorder*")

OR AB ("intellectual disabilit*" OR "developmental disabilit*" OR "Prader-Willi syndrome" OR "down syndrome" OR "fetal alcohol syndrome" OR "Fragile X" OR "Williams syndrome" OR "Smith-Magenis syndrome" OR "Cri-du-chat syndrome" OR "Angelman syndrome" OR "Rett syndrome" OR "autism spectrum disorder" OR ASD OR "asperger syndrome" OR autistic OR "communication disorder*" OR "learning disabilit*" OR "social communication disorder" OR "speech sound disorder" OR "brain injur*" OR "attention deficit disorder" OR ADHD OR "neurodevelopmental disorder*" OR "neurodevelopment disorder*")

S7. TI (dementia OR "Alzheimer disease" OR "Lewy body disease" OR "neurocognitive disorder*" OR "cognitive dysfunction" OR delirium)

OR AB (dementia OR "Alzheimer disease" OR "Lewy body disease" OR "neurocognitive disorder*" OR "cognitive dysfunction" OR delirium)

S8. S5 OR S6 OR S7

S9. S1 AND S4 AND S8

S10. S9 limited to publication year 2000 to current

Final set: S10 = 88 records

**Scopus**

**Information source:** Scopus

**Search platform:** Scopus

**Search date:** March 11, 2026

**Date range searched:** 2000 to current

**Limits and restrictions:** Publication year limited to 2000 to current

**Records retrieved:** 482 records

**Full search strategy**

TITLE-ABS-KEY (("restrictive practice*" OR restraint OR seclusion OR "isolation room*" OR coercion OR "coercive practice*" OR "forced treatment" OR "restrictive intervention*" OR "safety intervention*" OR "use of force" OR "chemical restraint*" OR "mechanical restraint*" OR "physical restraint*" OR "physical hold*" OR "holding intervention*" OR "liberty deprivation" OR "freedom restriction" OR "involuntary treatment" OR "compulsory treatment" OR "environmental restraint*" OR "pharmacological restraint*" OR "physical barrier restraint*")

AND

("real-time monitoring" OR "realtime monitoring" OR "real time monitoring" OR "continuous monitoring" OR "continuous observation" OR "continuous assessment" OR "remote monitoring" OR telemonitoring OR "digital monitoring" OR wearable* OR sensor* OR biosensor* OR "biometric monitoring" OR "patient monitoring" OR "health monitoring system*" OR "activity tracker*" OR "tracking device*" OR "continuous surveillance" OR "continuous tracking" OR "early warning system*" OR "predictive analytic*" OR "predictive model*" OR "prediction algorithm*" OR "predictive system*" OR "risk prediction" OR "risk forecasting" OR "early detection algorithm*" OR "forecasting model*" OR "clinical decision support" OR "decision support system*" OR "prognostic model*" OR "data mining" OR "pattern recognition" OR "artificial intelligence" OR "machine learning" OR "deep learning" OR "natural language processing" OR "neural network*" OR "large language model*" OR "generative AI" OR "generative artificial intelligence" OR chatbot* OR "conversational agent*" OR "embodied conversational agent*" OR "virtual assistant*" OR "digital assistant*" OR "intelligent assistant*" OR "relational agent*")

AND

("psychiatric disorder*" OR "mental disorder*" OR "psychiatric condition*" OR schizophrenia OR "post traumatic stress disorder" OR PTSD OR anxiety OR "anxiety disorder*" OR "psychotic disorder*" OR "mood disorder*" OR "psychological trauma" OR "mental health" OR "mental illness" OR psychiatric OR psychotic OR psychosis OR schizoph* OR "bipolar disorder*" OR depression OR mania* OR "trauma informed" OR "trauma inform*" OR "trauma focused" OR "trauma sensitive" OR "trauma aware*" OR "intellectual disabilit*" OR "developmental disabilit*" OR "Prader-Willi syndrome" OR "down syndrome" OR "fetal alcohol syndrome" OR "Fragile X" OR "Williams syndrome" OR "Smith-Magenis syndrome" OR "Cri-du-chat syndrome" OR "Angelman syndrome" OR "Rett syndrome" OR "autism spectrum disorder" OR ASD OR "asperger syndrome" OR autistic OR "communication disorder*" OR "learning disabilit*" OR "social communication disorder" OR "speech sound disorder" OR "brain injur*" OR "attention deficit disorder" OR ADHD OR "neurodevelopmental disorder*" OR "neurodevelopment disorder*" OR dementia OR "Alzheimer disease" OR "Lewy body disease" OR "neurocognitive disorder*" OR "cognitive dysfunction" OR delirium))

AND PUBYEAR > 1999

Final set: TITLE-ABS-KEY search combined with PUBYEAR > 1999 = 482 records.

**IEEE Xplore**

**Information source:** IEEE Xplore

**Search platform:** IEEE Xplore

**Search date:** March 11, 2026

**Date range searched:** 2000 to current

**Limits and restrictions:** Publication year limited to 2000 to current

**Records retrieved:** 24 records

**Full search strategy**

(

"All Metadata":"restrictive practice"

OR "All Metadata":restraint

OR "All Metadata":seclusion

OR "All Metadata":coercion

OR "All Metadata":"forced treatment"

OR "All Metadata":"chemical restraint"

OR "All Metadata":"mechanical restraint"

OR "All Metadata":"physical restraint"

OR "All Metadata":"use of force"

OR "All Metadata":"involuntary treatment"

OR "All Metadata":"compulsory treatment"

)

AND

(

"All Metadata":"real-time monitoring"

OR "All Metadata":"continuous monitoring"

OR "All Metadata":"remote monitoring"

OR "All Metadata":telemonitoring

OR "All Metadata":"digital monitoring"

OR "All Metadata":wearable

OR "All Metadata":sensor

OR "All Metadata":biosensor

OR "All Metadata":"patient monitoring"

OR "All Metadata":"health monitoring system"

OR "All Metadata":"early warning system"

OR "All Metadata":"predictive analytics"

OR "All Metadata":"predictive model"

OR "All Metadata":"prediction algorithm"

OR "All Metadata":"risk prediction"

OR "All Metadata":"clinical decision support"

OR "All Metadata":"artificial intelligence"

OR "All Metadata":"machine learning"

OR "All Metadata":"deep learning"

OR "All Metadata":"natural language processing"

OR "All Metadata":"neural network"

OR "All Metadata":chatbot

OR "All Metadata":"large language model"

OR "All Metadata":"generative AI"

OR "All Metadata":"conversational agent"

OR "All Metadata":"virtual assistant"

OR "All Metadata":"digital assistant"

OR "All Metadata":"relational agent"

)

AND

(

"All Metadata":"mental health"

OR "All Metadata":"psychiatric disorder"

OR "All Metadata":schizophrenia

OR "All Metadata":psychosis

OR "All Metadata":PTSD

OR "All Metadata":anxiety

OR "All Metadata":depression

OR "All Metadata":bipolar

OR "All Metadata":dementia

OR "All Metadata":delirium

OR "All Metadata":autism

OR "All Metadata":ADHD

OR "All Metadata":"intellectual disability"

OR "All Metadata":"developmental disability"

OR "All Metadata":"brain injury"

)

Final set: All Metadata search = 24 records.

**Note:** English-language eligibility will be applied during screening unless stated as a database limit.
