# Supplementary material for: Real-Time Monitoring and Predictive Analytics for Preventing and Reducing Restrictive Practices in Health and Social Care: Protocol for a Scoping Review
Source: JMIR Res Protoc. 2026 Nov 5;15:e98754. doi: 10.2196/98754 (PMC13739262; doi:10.2196/98754)
Supplement: Supplementary file 2 [file resprot-v15-e98754-s002.docx]

Title

**Real-time monitoring and predictive analytics to prevent and reduce restrictive practices in health and social care: a scoping review protocol**

Studies will be eligible where they examine a digital technology explicitly linked to restrictive-practice prevention, reduction, management, or avoidance through either a described care-response pathway or a reported restrictive-practice-related outcome. Restrictive practices within scope include physical restraint, mechanical restraint, chemical restraint, environmental restraint, and seclusion. Studies using alternative terminology will be considered where the described practice substantively involves restriction of movement, choice, autonomy, access, or liberty in a health or social care context.

**Appendix I: Table. Eligibility Criteria**

| **Inclusion criteria** | **Exclusion criteria** |
| --- | --- |
| **Population and setting:** Studies conducted in health or social care settings involving people who may be subject to, or at risk of being subject to, restrictive practices. Relevant settings may include aged care, disability support, mental health, acute care, community care, residential care, or other health and social care environments. | Studies conducted outside health or social care contexts, unless the intervention and findings are explicitly transferable to restrictive-practice reduction, prevention, management, avoidance, a restrictive-practice-related care response, or a restrictive-practice-related outcome in health or social care. |
| **Restrictive practices in scope:** Studies addressing one or more of the following restrictive practices: physical restraint, mechanical restraint, chemical restraint, environmental restraint, or seclusion. Equivalent terminology will be considered where the practice substantively involves restriction of a person’s movement, autonomy, access, liberty, or choice. | Studies that refer only generally to safety, risk, behavior, agitation, wandering, falls, surveillance, or monitoring without any explicit connection to restrictive practices, restraint, seclusion, or restrictive-practice-related care responses. |
| **Technology type:** Studies examining real-time monitoring, predictive analytics, artificial intelligence, machine learning, sensors, Internet of Things devices, digital decision-support systems, alert systems, dashboards, or related digital technologies used to identify, monitor, predict, prevent, reduce, manage, or respond to restrictive-practice risk. | Studies of digital technologies that have no explicit relationship to restrictive practices, restrictive-practice reduction, restrictive-practice prevention, or restrictive-practice-related outcomes. |
| **Minimum inclusion threshold:** To be included, the technology must be explicitly linked to either (a) a care response intended to prevent, reduce, manage, or avoid restrictive practices, or (b) a reported outcome related to restrictive practices. | Studies where the technology only detects, monitors, predicts, or classifies behavior, risk, movement, symptoms, or events without describing a care response related to restrictive-practice reduction or reporting a restrictive-practice-related outcome. |
| **Care response link:** Studies describing how technology outputs, such as alerts, predictions, sensor data, risk scores, or decision-support recommendations, inform staff action, care planning, de-escalation, non-restrictive alternatives, environmental adjustments, medication review, or other responses intended to reduce or prevent restrictive practices. | Studies reporting only technical performance, algorithm accuracy, usability, feasibility, or acceptability without explaining how the technology informs a restrictive-practice-related care response or outcome. |
| **Outcomes:** Studies reporting outcomes related to restrictive-practice prevention, reduction, frequency, duration, avoidance, substitution, escalation, de-escalation, staff response, care-process change, implementation outcome, service outcome, or clinical outcome connected to restrictive practices. | Studies reporting outcomes unrelated to restrictive practices, such as general safety, operational efficiency, user satisfaction, diagnostic performance, or general behavioral monitoring, unless a restrictive-practice link is clearly stated. |
| **Study designs and evidence sources:** Empirical studies, implementation studies, evaluations, pilot studies, feasibility studies, mixed methods studies, qualitative studies, and quantitative studies that provide sufficient information about the technology, setting, restrictive-practice link, care response, or outcome. Systematic reviews may be included where they independently meet the eligibility criteria and provide relevant information about technologies, care-response pathways, implementation processes, or outcomes. Primary studies represented in included systematic reviews will be charted separately only where they independently meet the eligibility criteria and provide additional relevant information. | Commentaries, editorials, opinion pieces, protocols, abstracts, or reports that do not provide sufficient methodological or conceptual detail to determine whether the technology meets the minimum inclusion threshold. Duplicate or overlapping outcome information already represented in another included source will not be counted more than once. |
| **Publication status and source type:** Peer-reviewed sources will be included where they meet the review scope and provide enough information for charting. | Sources that cannot be accessed in sufficient detail for eligibility assessment or data charting. |
| **Language:** Only studies in English will be considered for inclusion. | Studies that are not in English will be excluded. |
| **Multiple reports from the same study:** Multiple reports may be included where they provide distinct information about the technology, implementation process, care response, restrictive-practice type, evaluation component, or outcome. Reports from the same study should be linked during charting. | Duplicate reports that do not add new information relevant to the technology, care response, restrictive-practice outcome, or implementation process. |

**Notes:** The minimum inclusion threshold is therefore not the presence of a digital technology alone, but the presence of an explicit link between the technology and restrictive-practice reduction, prevention, management, care response, or outcome.
