# Supplementary material for: Real-Time Monitoring and Predictive Analytics for Preventing and Reducing Restrictive Practices in Health and Social Care: Protocol for a Scoping Review
Source: JMIR Res Protoc. 2026 Nov 5;15:e98754. doi: 10.2196/98754 (PMC13739262; doi:10.2196/98754)
Supplement: Supplementary file 3 [file resprot-v15-e98754-s003.docx]

Title

**Real-time monitoring and predictive analytics to prevent and reduce restrictive practices in health and social care: a scoping review protocol**

*The charting form is organized to align with the three review subquestions. Tables 1-3 primarily support Q1 by capturing source, population, setting, technology, model, and data-source characteristics. Tables 4-7 primarily support Q2 by capturing care-response pathways, implementation actions, mechanisms, and reported outcomes. Tables 8-9 primarily support Q3 by capturing implementation determinants, barriers, enablers, and ethical or human-rights considerations. Tables 10-11 support annotation, data management, and auditability.*

*Instructions: Complete one form per included record. Use exact wording from the article where helpful and note page numbers for audit. Where lists are provided in brackets, select or enter the appropriate option(s).*

## Table 1. Citation and study identifiers

| Full citation (Author, Year, Title, Journal) |  |
| --- | --- |
| DOI / PMID |  |
| Country |  |
| Funding / conflicts (optional) |  |
| Corresponding care setting (see list) |  |
| Study design / approach |  |
| Study period / timeframe |  |
| Unit of charting (source; study; intervention component; implementation component; evaluation component; other) |  |
| Linked reports / companion publications (Record IDs or citations of related reports from the same study, intervention, or implementation program) |  |
| PDF filename / record ID |  |

## Table 2. PCC and study characteristics

| Population & n (diagnostic group; cohort size) |  |
| --- | --- |
| Age group (Child/Adolescent; Adult; Older adult; Mixed) |  |
| Care setting (Residential aged care; Acute psychiatry; Disability support; Hospital; Community care; ED) |  |
| Restrictive-practice type(s) (Physical restraint; Mechanical restraint; Chemical restraint; Environmental restraint; Seclusion; Other/equivalent; Not clearly specified) |  |
| Restrictive-practice terminology used by authors (Extract exact term(s), with page number) |  |
| Minimum inclusion threshold met? (Yes: linked to care response; Yes: restrictive-practice outcome reported; Yes: both care response and outcome; Unclear; No) |  |
| Implementation status reported by source (Development; Feasibility/pilot; Implemented in routine care; Evaluation; Scale-up; Unclear) |  |
| Evidence for implementation status (exact wording and page number) |  |
| Context notes (e.g., ward type, geography) |  |
| Ethics approvals / consent model (optional) |  |
| Eligibility rationale / screening note (Briefly state how the source links the technology to restrictive-practice prevention, reduction, management, care response, or outcome, with page number where available.) |  |

## Table 3. Technology description

| Technology type/modality (Sensor; wearable; camera/video; ambient/environmental; bed/chair; audio/acoustic; electronic health record (EHR) log‑derived; multimodal; decision support; dashboard; alert system; AI model; predictive analytics; other) |  |
| --- | --- |
| Monitoring or predictive function (Monitoring only; Predictive only; Both; Unclear) |  |
| AI/model subtype, if applicable (Random forest; LSTM; neural network; decision tree; regression; rule-based; NLP/LLM; other; not stated) |  |
| Primary data source(s) (Sensor stream; EHR; behavioral record; medication record; incident report; staff observation; multimodal; other; unclear) |  |
| Technology output (Alert; risk score; classification; prediction; dashboard display; recommendation; notification; other) |  |
| Behavior of concern (e.g., aggression; wandering; agitation) |  |
| Sensor/Modality details (signals, sampling, vendor, etc.) |  |
| Placement/Location (On body: wrist; chest; belt; Fixed: room; corridor; bed/chair) |  |
| Algorithm or technical performance (if predictive: AUC, Sens/Spec, PPV/NPV) |  |
| Data flow/integration (EHR; dashboards; standalone; mobile app; staff workflow; other; not stated) |  |
| Privacy/ethics notes (e.g., consent, governance) |  |
| Key quotation(s) (with page) |  |

Note: NLP/LLM applications should be charted only where they meet the minimum inclusion threshold.

## Table 4. Care-response pathway following detection or prediction

*Complete one action record per distinct implementation action (ERIC).*

| Action ID | Trigger or technology output (Alert; risk score; prediction; classification; dashboard display; recommendation; notification; other) | ERIC strategy (Free text or controlled ERIC term) | ERIC category (Parent category) | Care response linked to technology output (Staff check; de-escalation; behavior support; environmental adjustment; medication review; care-plan review; family/carer contact; clinical escalation; non-restrictive alternative; other) | Care-response link evidence status (Explicitly reported; interpreted or proposed by source authors; unclear; not reported) | Restrictive-practice reduction intent (Explicit; implied; not stated) | Restrictive-practice pathway (Prevention; reduction; avoidance; substitution; escalation prevention; monitoring only; unclear) | Human decision point (Staff judgment; clinical protocol; automated recommendation; multidisciplinary review; unclear) | Delivery channel (Dashboard; mobile alert; EHR notification; verbal handover; written care plan; other) | Recipient role(s) (Nurse; care worker; clinician; manager; family/carer; multidisciplinary team; other) |
| --- | --- | --- | --- | --- | --- | --- | --- | --- | --- | --- |
|  |  |  |  |  |  |  |  |  |  |  |

## Table 5. Implementation strategy, mechanism and audit details

| Action ID | Decision rule/Protocol (Free text; include page number) | Automation level (Manual; semi-automated; automated; unclear) | Dose/Frequency (As reported) | Integration with EHR/workflow (Yes; no; partial; unclear; details) | Fidelity/Adherence (As reported; not reported) | Action latency (Minutes/hours/days; not reported) | Reported or source-author-proposed mechanism of action (Technical; behavioral; both; other; unclear; not reported) | Evidence status (Explicitly reported, with source page or quotation; interpreted or proposed by source authors; unclear; not reported) | Linked outcome ID (O1, O2, O3, not linked) |
| --- | --- | --- | --- | --- | --- | --- | --- | --- | --- |
|  |  |  |  |  |  |  |  |  |  |

## Table 6. Implementation outcomes

*Repeat rows as needed.*

| Outcome label | Outcome metric | Unit/Denominator | Baseline value, if reported | Follow‑up value, if reported | Reported direction of change (↑/↓/↔/NR) | Variance or confidence interval, if reported | Notes (page) |
| --- | --- | --- | --- | --- | --- | --- | --- |
|  |  |  |  |  |  |  |  |

## Table 7. Service, clinical, and restrictive-practice outcomes

*Repeat rows as needed.*

| Outcome label | Outcome metric | Outcome directly linked to technology? (Yes; No; Unclear) | Unit / Denominator | Baseline value, if reported | Follow‑up value, if reported | Reported direction of change (↑/↓/↔/NR) | Variance or confidence interval, if reported | Restrictive-practice outcome category (Frequency; duration; reduction; prevention; avoidance; escalation/de-escalation; substitution; PRN psychotropic use; seclusion events; restraint rate; other) | Restrictive-practice type linked to outcome (Physical; mechanical; chemical; environmental; seclusion; mixed; unclear) | Notes (page) |
| --- | --- | --- | --- | --- | --- | --- | --- | --- | --- | --- |
|  |  |  |  |  |  |  |  |  |  |  |

## Table 8. Implementation determinants, barriers, and enablers

*Code each issue and indicate whether it was addressed. Repeat rows as needed.*

| Issue ID | Determinant statement (quote + page) | Determinant bin(s) (Inner; Outer; Individual; Intervention) | Polarity (Barrier/Enabler/Mixed) | Theme (e.g., connectivity; interoperability; workflow fit) | Impact (High/Moderate/Low) | Addressed? (Yes/No/Partially/Not stated) | Linked ERIC strategy | Reported mechanism associated with determinant or strategy |
| --- | --- | --- | --- | --- | --- | --- | --- | --- |
|  |  |  |  |  |  |  |  |  |

## Table 9. Ethical and human-rights considerations

| Issue ID | Residual issue | Evidence source | Ethical issue type (Stigma; coercion; bias; surveillance harms; autonomy; privacy; equity; consent; governance; human-rights framing; restrictive-practice normalization; other) | Ethical framing (Rights-based; risk management; safety-focused; behavior-control framing; person-centered; unclear) | Restrictive-practice normalization risk (Yes; No; Unclear; Not applicable) | Mitigation described? (Yes; No; Partial; Not applicable) | Affected group (Older people; people with dementia; people with disability; mental health consumers; staff; family/carers; other; not stated) | Privacy/Ethics flag | Reviewer note (Extract exact quotation and page number) |
| --- | --- | --- | --- | --- | --- | --- | --- | --- | --- |
|  |  |  |  |  |  |  |  |  |  |

## Table 10. Additional notes or annotations

Use for clarifications, classification rationale, and cross-references to figures/tables.

## Table 11. Data management and audit trail

| Extractor (name/initials) |  |
| --- | --- |
| Second reviewer (check) |  |
| Extraction date (YYYY‑MM‑DD) |  |
| Eligibility and charting decision verified? (Yes; No) |  |
| Disagreements / resolutions |  |
| Final resolution reviewer, if applicable |  |
| Duplicate information checked? (Yes; No; Not applicable) |  |
| Duplicate or companion report? (Yes; No; Unclear) |  |
| Linked-report reconciliation completed? (Yes; No; Not applicable) |  |
| Linked record IDs |  |
| Primary report for synthesis? (Yes; No; Not applicable) |  |
| Codebook version used |  |
