# Supplementary material for: Real-Time Monitoring and Predictive Analytics for Preventing and Reducing Restrictive Practices in Health and Social Care: Protocol for a Scoping Review
Source: JMIR Res Protoc. 2026 Nov 5;15:e98754. doi: 10.2196/98754 (PMC13739262; doi:10.2196/98754)
Supplement: Supplementary file 4 [file resprot-v15-e98754-s004.docx]

Title

**Real-time monitoring and predictive analytics to prevent and reduce restrictive practices in health and social care: a scoping review protocol**

**PRISMA-P 2015 Checklist Adapted for This Scoping Review Protocol**

*Notes. PRISMA-P has 17 numbered items (26 including subitems). Checklist wording is reproduced from PRISMA-P 2015 under CC BY 4.0. “Not applicable” is used where an item does not apply to this scoping review protocol.*

| **Section and topic** | **Item No** | **PRISMA-P checklist item** | **Location where item is reported** |
| --- | --- | --- | --- |
| **ADMINISTRATIVE INFORMATION** | | | |
| Identification | 1a | Identify the report as a protocol of a systematic review | Page 1, Title. |
| Update | 1b | If the protocol is for an update of a previous systematic review, identify as such | Not applicable. |
| Registration | 2 | If registered, provide the name of the registry (such as PROSPERO) and registration number | Pages 1 and 4, Abstract—Results; Protocol Registration and Search Chronology; reference 38. |
| Contact | 3a | Provide name, institutional affiliation, e-mail address of all protocol authors; provide physical mailing address of corresponding author | Page 1, Author list, affiliations, and Corresponding Author. |
| Contributions | 3b | Describe contributions of protocol authors and identify the guarantor of the review | Page 9, Authors’ Contributions. |
| Amendments | 4 | If the protocol represents an amendment of a previously completed or published protocol, identify as such and list changes; otherwise, state plan for documenting important protocol amendments | Pages 4 and 6, Protocol Registration and Search Chronology; Protocol Amendments. |
| Sources | 5a | Indicate sources of financial or other support for the review | Page 9, Funding: No financial support. |
| Sponsor | 5b | Provide name for the review funder and/or sponsor | No sponsor. |
| Role of sponsor or funder | 5c | Describe roles of funder(s), sponsor(s), and/or institution(s), if any, in developing the protocol | Not applicable. |
| **INTRODUCTION** | | | |
| Rationale | 6 | Describe the rationale for the review in the context of what is already known | Pages 2-3, Introduction. |
| Objectives | 7 | Provide an explicit statement of the question(s) the review will address with reference to participants, interventions, comparators, and outcomes (PICO) | Pages 1 and 3, Abstract—Objective; Objectives and Review Questions. |
| **METHODS** | | | |
| Eligibility criteria | 8 | Specify the study characteristics (such as PICO, study design, setting, time frame) and report characteristics (such as years considered, language, publication status) to be used as criteria for eligibility for the review | Page 4, Eligibility Criteria; Multimedia Appendix 2. |
| Information sources | 9 | Describe all intended information sources (such as electronic databases, contact with study authors, trial registers or other grey literature sources) with planned dates of coverage | Pages 4-5, Information Sources; Search Strategy; Multimedia Appendix 1. |
| Search strategy | 10 | Present draft of search strategy to be used for at least one electronic database, including planned limits, such that it could be repeated | Page 5, Search Strategy; Multimedia Appendix 1. |
| Data management | 11a | Describe the mechanism(s) that will be used to manage records and data throughout the review | Page 5, Source Selection; Data Extraction and Charting; Multimedia Appendix 3. |
| Selection process | 11b | State the process that will be used for selecting studies (such as two independent reviewers) through each phase of the review (that is, screening, eligibility and inclusion in meta-analysis) | Page 5, Source Selection. |
| Data collection process | 11c | Describe planned method of extracting data from reports (such as piloting forms, done independently, in duplicate), any processes for obtaining and confirming data from investigators | Page 5, Data Extraction and Charting; Multimedia Appendix 3. |
| Data items | 12 | List and define all variables for which data will be sought (such as PICO items, funding sources), any pre-planned data assumptions and simplifications | Pages 5-6, Data Extraction and Charting; Analytical Framework; Multimedia Appendix 3. |
| Outcomes and prioritization | 13 | List and define all outcomes for which data will be sought, including prioritization of main and additional outcomes, with rationale | Pages 5-6, Data Extraction and Charting; Synthesis of Results. |
| Risk of bias in individual studies | 14 | Describe anticipated methods for assessing risk of bias of individual studies, including whether this will be done at the outcome or study level, or both; state how this information will be used in data synthesis | Page 5, Critical Appraisal. |
| Data synthesis | 15a | Describe criteria under which study data will be quantitatively synthesised | Page 6, Quantitative Summarization and Qualitative Synthesis. |
|  | 15b | If data are appropriate for quantitative synthesis, describe planned summary measures, methods of handling data and methods of combining data from studies, including any planned exploration of consistency (such as I², Kendall’s τ) | Not applicable. |
|  | 15c | Describe any proposed additional analyses (such as sensitivity or subgroup analyses, meta-regression) | Not applicable. |
|  | 15d | If quantitative synthesis is not appropriate, describe the type of summary planned | Page 6, Quantitative Summarization and Qualitative Synthesis; Data Presentation. |
| Meta-bias(es) | 16 | Specify any planned assessment of meta-bias(es) (such as publication bias across studies, selective reporting within studies) | Not applicable. |
| Confidence in cumulative evidence | 17 | Describe how the strength of the body of evidence will be assessed (such as GRADE) | Not applicable. |

**References**

1. Moher D, Shamseer L, Clarke M, et al. Preferred reporting items for systematic review and meta-analysis protocols (PRISMA-P) 2015 statement. Syst Rev. 2015;4(1):1. doi:10.1186/2046-4053-4-1.

2. Shamseer L, Moher D, Clarke M, et al. Preferred reporting items for systematic review and meta-analysis protocols (PRISMA-P) 2015: elaboration and explanation. BMJ. 2015;349:g7647. doi:10.1136/bmj.g7647.
